# The Nitrogen Contribution of Different Plant Parts to Wheat Grains: Exploring Genotype, Water, and Nitrogen Effects

**DOI:** 10.3389/fpls.2016.01986

**Published:** 2017-01-09

**Authors:** Rut Sanchez-Bragado, M. Dolors Serret, José L. Araus

**Affiliations:** Plant Physiology Department, University of BarcelonaBarcelona, Spain

**Keywords:** nitrogen content, nitrogen isotope composition, ear, grains, wheat

## Abstract

The flag leaf has been traditionally considered as the main contributor to grain nitrogen. However, during the reproductive stage, other organs besides the flag leaf may supply nitrogen to developing grains. Therefore, the contribution of the ear and other organs to the nitrogen supplied to the growing grains remains unclear. It is important to develop phenotypic tools to assess the relative contribution of different plant parts to the N accumulated in the grains of wheat which may helps to develop genotypes that use N more efficiently. We studied the effect of growing conditions (different levels of water and nitrogen in the field) on the nitrogen contribution of the spike and different vegetative organs of the plant to the grains. The natural abundance of δ^15^N and total N content in the flag blade, peduncle, whole spike, glumes and awns were compared to the δ^15^N and total N in mature grains to trace the origin of nitrogen redistribution to the grains. The δ^15^N and total N content of the different plant parts correlated positively with the δ^15^N and total N content of mature grains suggesting that all organs may contribute a portion of their N content to the grains. The potential contribution of the flag blade to grain N increased (by 46%) as the growing conditions improved, whereas the potential contribution of the glumes plus awns and the peduncle increased (46 and 31%, respectively) as water and nitrogen stress increased. In general, potential contribution of the ear providing N to growing grains was similar (42%) than that of the vegetative parts of the plants (30–40%), regardless of the growing conditions. Thus, the potential ear N content could be a positive trait for plant phenotyping, especially under water and nitrogen limiting conditions. In that sense, genotypic variability existed at least between old (tall) and modern (semidwarf) cultivars, with the ear from modern genotypes exhibiting less relative contribution to the total grain N. The combined use of δ^15^N and N content may be used as an affordable tool to assess the relative contribution of different plant parts to the grain N in wheat.

## Introduction

In terrestrial ecosystems, nitrogen is often the most limiting element in plant growth (Vitousek, 1994) and alongside drought stress, a lack of nitrogen can limit crop quality and productivity (Passioura, 2002). In particular, water availability can affect crop growth, soil nitrogen dynamics and the utilization of plant nitrogen from soil and fertilizers (Raimanová and Haberle, 2010). In addition, grain filling and by extension grain yield is dependent on carbon and nitrogen metabolism (Zhang et al., 2010). The nitrogen requirements of developing grains in wheat are mainly supplied by degradation of proteins derived from different plant organs (Dalling et al., 1976; Simpson et al., 1983; Bancal, 2009). Cereals may accumulate most of their nitrogen in vegetative organs before ear emergence and then redistribute it during grain development (Dalling et al., 1976; Hortensteiner, 2002). Nevertheless, even though an additional source of N may be taken up by roots directly from the soil and assimilated between anthesis and physiological maturity (Dupont and Altenbach, 2003), the greatest proportion of N in the grain is redistributed from the vegetative parts (Perez et al., 1989; Jukanti et al., 2008). In fact, N uptake after anthesis is considered in most cases to be minimal (Perez et al., 1989). Moreover, grain filling is the period when the nitrogen content of different plant parts is substantially reduced (Lopes et al., 2006) as a consequence of protein hydrolysis, which remobilizes amino acids for export to developing grains (Feller and Fischer, 1994).

Traditionally, the flag leaf has been considered as the main contributor to grain nitrogen due to its large protein content (Millard and Grelet, 2010). Indeed, during the last decade, most of the studies dealing with N accumulation in grains have only focused on leaf lamina nitrogen (Hortensteiner, 2002; Bahrani and Joo, 2010). However, during the reproductive stage, as sink strength (grain N) increases after anthesis, other organs besides the flag leaf may become organs of nitrogen supply to developing grains (Waters et al., 1980). Thus, apart from the flag leaf blade, the contribution of the ear as well as the lower parts of the plant may be relevant. Ear photosynthesis is considered as an important source of assimilates for grain filling in wheat and other cereals (Araus et al., 1993; Bort et al., 1994; Tambussi et al., 2007; Sanchez-Bragado et al., 2014a,b) especially under drought conditions (Tambussi et al., 2005). To date, several studies have analyzed the photosynthetic contribution of the ear to grain filling (Tambussi et al., 2007; Maydup et al., 2010, 2012, 2014). Nevertheless, the ear's contribution in terms of nitrogen supply to growing grains still remains unclear, despite studies that have emphasized its importance (Simpson et al., 1983; Lopes et al., 2006). Thus, contribution of other parts of the spike such as the glumes and awns to grain nitrogen might be relevant because these tissues have the longest period of metabolic activity during grain filling (Simpson et al., 1983; Jukanti et al., 2008; Bahrani and Joo, 2010). In fact, measurements of enzyme activities related to nitrogen metabolism (i.e., glutamine synthetase and glutamate dehydrogenase) in floral parts of wheat (awns, glumes) and the flag leaf reveal the existence of ammonia turnover activity in all these organs (Maheswari et al., 1992), suggesting that glumes and awns in addition to the flag leaf can play an important role in nitrogen metabolism during grain filling. For instance, the relative daily contribution of different organs to N accumulation in wheat grains during mid grain filling has been reported as 40% from leaves (leaf lamina and sheath), 23% from glumes, 23% from stems, and 16% from roots (Simpson et al., 1983). However, in advanced stages of grain development, the role of the glumes in terms of contribution to grain nitrogen has been observed to be greater (38%) than in the flag leaf (19%) (Lopes et al., 2006). Thus, protein content in the flag leaf blade seems to be constant until anthesis but reduces during grain filling, whereas glumes can accumulate proteins until 5 days after anthesis (Waters et al., 1980). Thus, glumes can act as a temporal sink for nitrogen in the absence of alternative sinks prior to rapid grain filling (Dalling et al., 1976). Then, during rapid grain filling as the sink strength of the grain increases, glumes are converted into a nitrogen source, presumably remobilizing their accumulated nitrogen to the grains (Waters et al., 1980; Lopes et al., 2006). In contrast, the nitrogen contribution of the stem to grain nitrogen has been observed to be minor due to its low protein content available for mobilization (and low N content loss) compared to the glumes and the flag leaf (Waters et al., 1980). However it may be relevant to study the potential contribution of this plant part since all the N that leaves remobilize to the grains has to pass through that plant part, especially the upper part of the stem (peduncle). This is particularly important for the leaves below the flag leaf since start to senesce earlier (compared with the flag leaf) during grain filling. Thus, it is important to have a better understanding of the role of the spike as a source of N to growing grains, in order to develop phenotypic tools to assess the relative contribution of different plant parts to the N accumulated in the grains of wheat. Long term, the objective is to develop genotypes that use N more efficiently.

The natural variation of the stable nitrogen isotopes (^15^N/^14^N) has been considered as a tool to study nitrogen plant dynamics and as a tracer of the nitrogen sources used by the plant (Evans, 2001; Rossato, 2002; Malagoli et al., 2005). Although it is known that plant nitrogen isotope abundance (δ^15^N) is linked to nitrogen metabolism (Robinson, 2000; Ellis, 2002; Pritchard and Guy, 2004), underlying biochemical mechanisms that affect nitrogen isotope composition (δ^15^N) are not yet completely understood (Cernusak et al., 2009). One of the reasons for this might be the fractionation that the nitrogen isotope undergo during enzymatic assimilation of ammonium or nitrate into other forms (Yousfi et al., 2012). Besides, other processes such as volatilization, translocation, or nitrogen recycling in the plant can discriminate positively or negatively against ^15^N (Robinson et al., 1998; Evans, 2001). Nevertheless, in spite of the discrimination processes affecting δ^15^N (Evans, 2001), vegetative organs in wheat have been reported to exhibit different δ^15^N (Lopes et al., 2006; Yousfi et al., 2009, 2013). Furthermore, the natural abundance of the stable N isotopes has been observed to be affected by water availability (Handley et al., 1994; Robinson, 2000; Lopes et al., 2004, 2006; Araus et al., 2013) and the nitrogen source (Cliquet et al., 1990). Thus, providing that nitrogen isotope fractionation from the vegetative organs to the growing grains is negligible (or at least constant; Dawson et al., 2002; Serret et al., 2008), an alternative method to study N uptake and remobilization by the plant could be to compare the δ^15^N in its natural abundance in the different plant organs during grain filling.

The aim of this work is to study the contribution of different plant parts to the nitrogen in grains under different growing conditions (using different water and nitrogen levels) in a set of old (i.e., tall) and modern (i.e., semidwarf) durum wheat genotypes. To this end, the natural abundance of δ^15^N and the total N content in the flag leaf blade, peduncle, roots, the whole spike and different tissues (glumes and awns) of the plant were compared to values of δ^15^N and total N content in mature grains in order to trace the origin of the nitrogen redistributed into the grains. The final objective is to assess the performance of the total N content together with the δ^15^N in its natural abundance as an affordable tool to assess the relative contribution of different plant parts to the N accumulated in the grains of wheat.

## Materials and methods

### Germplasm used and experimental conditions

Ten durum wheat [*Triticum turgidum* L. ssp. *durum* (Desf.) Husn.] genotypes were studied: five old Spanish cultivars (*Blanqueta, Griego de Baleares, Negro, Jerez 37*, and *Forment de Artes*) and five modern (i.e., semidwarf) Spanish cultivars delivered after 1990 (*Anton, Bolo, Don Pedro, Regallo*, and *Sula*). Old cultivars were chosen based on their similarity to the phenology of modern cultivars. Field experiments were conducted during two growing seasons, one in 2011 and the other in 2012 (Sanchez-Bragado et al., 2014a), at the experimental station of the Instituto Nacional de Investigación y Tecnología Agraria y Alimentaria (INIA) in Aranjuez (40°03′N, 3°31′E, 500 m asl). In the experimental field, soil is Entisol Fluvent Xerofluvent, with the upper 0.4 m having an organic matter content of 4.9 g/kg, total nitrogen content of 0.37 g/kg, carbonate content of 233 g/kg, pH of 8.1 and electric conductivity of 0.164 dS/m (Araus et al., 2013). During 2012 the five old Spanish genotypes grown under support irrigation conditions were also discarded due to lodging. Two water treatments (support irrigation, SI, and rain-fed, RF) combined with two nitrogen regimes (fertilized, HN, and non-fertilized, LN) were assayed. The trials were planted on 30 December 2010 and 18 November 2011 (from now on designated by their harvest year 2011 and 2012, respectively) in plots with six rows 0.20 m apart, covering a total area of 7.1 m^2^ (5 m length and 1.42 m width) per plot. Total accumulated precipitation during the 2011 and 2012 seasons was 275.4 and 126.1 mm, respectively. For both years sprinkler irrigation was applied to irrigated plots around initiation of booting (beginning of April) and grain filling (around May 15th and 30th) with ~60 mm of water on each date. Prior to sowing, all trials received 60 kg ha^−1^ of phosphorous as superphosphate (18%) and 60 kg ha^−1^ potassium as potassium chloride (60%). Further, the HN plants were dressed with nitrogen applied at the beginning of tillering (January 27th in 2011 and December 29th in 2012) and jointing (March 20th in 2011 and February 20th in 2012) using a dose of 45 and 105 kg ha^−1^ of urea (46%), respectively. The LN plants were not N fertilized, relying exclusively on the N availability in the soil before sowing. Water and nitrogen treatments were arranged according to a split-split plot design with three replicates. Experiment plots were kept free of weeds, insect pests, and diseases by recommended chemical measures (Sanchez-Bragado et al., 2014a).

Phenology was recorded throughout the growth cycle (Zadoks et al., 1974). Sampling was performed around 7 days after anthesis (7th May) in 2011 and 10 days after anthesis (18th April) in 2012. In 2011 stomatal conductance (g_s_) was measured with a leaf porometer (Decagon; Pullman, USA) in one leaf per plot at the mid point of grain filling. Similarly, chlorophyll content was measured with a SPAD-502 Minolta chlorophyll meter (Spectrum Technologies, Plainfield, IL, USA). In 2011, roots were collected from the upper layer (0–10 cm) with a split tube (Eijkelkamp Soil & Water, The Netherlands), rinsed with distilled water and then placed inside a paper envelope. Based on similar values of GY obtained from previous studies two old Spanish cultivars (*Blanqueta* and *Negro*) and two modern cultivars (*Anton* and *Bolo*) with three replicates for each growing condition (4 treatments) were selected for root extraction (48 plots). Thereafter, five representative flag leaves, peduncle and ears were collected per plot, and oven dried together with the collected roots at 70°C for 48 h. Once dried, in 2011 the entire spike and flag leaf blade were weighed and ground, whereas in 2012 the glumes, awns, flag leaf blade, and peduncle were separated after drying, weighed, and finely ground for total nitrogen content and nitrogen isotope signature analyses as described below. In addition, based on similar grain nitrogen contents obtained in previous studies, developing grains of a modern cultivar (*Regallo*) and an old Spanish cultivar (*Jerez 37*) from the same set of samples (with four growing conditions and three replicates per genotype) were also separated, weighed and finely ground for total nitrogen content and nitrogen isotope analyses as explained below. At maturity, the central four rows of each plot were harvested and grain yield (GY) recorded. Harvesting was performed manually and by machine in 2011 and 2012, respectively. Subsequently mature grains were processed for N content and isotope analysis. Total nitrogen GY was then calculated as the product of GY by N content on a dry matter basis of mature grains.

### Nitrogen concentration and stable isotope composition

The total N content and stable nitrogen isotope signature in the dry matter of the entire spike, glumes, awns, flag leaf, peduncle, roots, developing, and mature grains were analyzed. Approximately 1 mg of each sample was weighed into tin capsules and measured with an elemental analyser (Flash 1112 EA; ThermoFinnigan, Bremen, Germany) coupled with an Isotope Ratio Mass Spectrometer (Delta C IRMS, ThermoFinnigan, Bremen, Germany) operating in continuous flow mode in order to determine the total N content and the stable nitrogen (^15^N/^14^N) isotope ratios. The (^15^N/^14^N) ratios of plant material were expressed in δ notation (Coplen, 2008): δ^15^N = (^15^N/^14^N)_sample_/(^15^N/^14^N)_standard_ − 1, where “sample” refers to plant material and “standard” N_2_ in air.

### Water-soluble fraction

The protein-free water-soluble fractions (WSFs) of the flag leaf and spike (entire spike, awns, and glumes) were extracted from the same dry samples tested for nitrogen isotopes, as described previously (Cabrera-Bosquet et al., 2011; Yousfi et al., 2013). Summarizing, 50 mg of either fi ne leaf or ear powder were suspended with 1 ml of MilliQ water in an Eppendorf tube (Eppendorf Scientific, Hamburg, Germany) for 20 min at about 5°C. After centrifugation (12,000 g for 5 min at 5°C), the pellet was discarded and the supernatant containing the WSF was heated at 100°C for 3 min, where the heat-denatured proteins precipitated. Subsequently, samples were centrifuged again (12,000 g for 5 min at 5°C) to separate previously denatured proteins from the soluble fraction. Aliquots of 40 μl of supernatant containing protein-free WSF were transferred into tin capsules for nitrogen analysis. The capsules containing the aliquots were oven dried at 60°C.

### Total organ N and potential, relative organ N contribution to grain N

Total organ nitrogen content of the flag leaf blade, peduncle, whole spike, glumes, and awns was calculated as the product of nitrogen content on a dry matter basis in the different organs multiplied by their respective dry weight. For the whole spike N calculation, the total grain N of developing grains was subtracted from the calculation (taking into account the treatment and genotype). The potential organ nitrogen contributions of the flag leaf blade, peduncle, spike, glumes, and awns to the nitrogen accumulated in the grains were calculated as the product of N content of each organ multiplied by its respective dry weight and standardized (i.e., divided) by the total N content of mature grains per spike. The relative contribution of the different organs was calculated as the potential organ N contribution for the specific organ divided by the sum of the potential organ N of all organs studied. In addition the ratio between mean values of dry weight (g) under rainfed (RF) vs. supplemental irrigation (SI) was calculated for the spike, flag leaf, peduncle, glumes, and awns.

### Statistical analysis

Treatment, organ, and genotype effects were assessed by means of Analysis of Variance (ANOVA). Water regime, nitrogen supply, organ and their interactions were included as fixed factors. Means were compared by Tukey's HSD test. A bivariate correlation procedure was constructed to analyse the relationships between the measured traits. Statistical analyses were performed using the SPSS 18.0 statistical package (SPSS Inc., Chicago, IL, USA). Figures were created using Microsoft Excel 2010 (Microsoft Corporation).

## Results

### Grain yield and organ nitrogen content

Average grain yield (GY) and nitrogen grain yield (nitrogen GY) across treatments and genotypes were lower in 2011 (1.7 Mg·ha^−1^) than in 2012 (3.1 Mg·ha^−1^; Tables 1, 2, respectively). However, the interaction of nitrogen and water showed an effect on GY in the two-way ANOVA analysis (Table S1). Considering the different treatments, the highest average GY and nitrogen GY was observed under supplemental irrigation, regardless of the N fertilization (fertilized, SI+HN or non-fertilized, SI-LN) conditions for both 2011 (Table 1) and 2012 (Table 2). Modern cultivars showed higher GY and nitrogen GY than the old cultivars during both growing seasons (Table S1).

**Table 1:** **Genotype (G), water supply (W), nitrogen (N), and organ (O) effects (ANOVA) and mean values and of nitrogen isotope composition (δ^15^N) in the water-soluble fraction (WSF) and dry matter (DM) of the flag leaf blade and entire spike as well as in mature grains and roots, nitrogen content (N content), total nitrogen content per organ (Total organ N), grain yield (GY) and total nitrogen content of mature grains per spike (Grain N·spike^−1^)**.

	**Mean Values**				**ANOVA**			
	**SI+HN**	**SI-LN**	**WS+HN**	**WS-LN**	**G**	**W**	**N**	**O**
**ROOTS**								
δ^15^N DM (‰)	0.94^b^	4.78^c^	−0.84^a^	4.06^c^	ns	ns	^***^	−
**FLAG LEAF**								
N content (%DM)	3.98^c^	2.85^a^	3.67^b^	2.85^a^	ns	^**^	^***^	−
Total organ N (mg)	4.22^a^	3.29^a^	4.34^a^	3.36^a^	ns	ns	ns	−
δ^15^N DM (‰)	1.29^a^	6.68^b^	1.02^a^	6.06^b^	ns	ns	^***^	−
δ^15^N WSF (‰)	0.53^a^	6.20^b^	−0.09^a^	5.67^b^	ns	ns	^***^	−
**SPIKE**								
N content (%DM)	1.79^b^	1.48^a^	1.73^b^	1.49^a^	^***^	ns	^***^	−
Total organ N (mg)	12.38^ab^	10.49^a^	12.83^b^	11.09^ab^	ns	^*^	^*^	−
δ^15^N DM (‰)	1.61^b^	6.15^c^	0.86^a^	5.51^c^	ns	^**^	^***^	−
δ^15^N WSF (‰)	3.21^b^	7.68^d^	2.44^a^	7.05^c^	ns	^**^	^***^	−
**GRAIN**								
N content (%DM)	2.54^c^	1.81^a^	2.93^d^	2.18^b^	^*^	^***^	^***^	−
Grain N·spike^−1^	24.41^a^	21.38^a^	21.66^a^	20.94^a^	^**^	ns	ns	−
δ^15^N DM (‰)	3.36^b^	6.58^d^	2.26^a^	6.18^c^	ns	^***^	^***^	−
GY (Mg·ha^−1^)	2.31^c^	1.87^b^	1.26^a^	1.41^a^	ns	^***^	^**^	−
Nitrogen GY (Mg·ha^−1^)	0.06^c^	0.03^ab^	0.04^b^	0.03^a^	ns	^***^	^***^	−
N content (%DM)	−	−		−	−	−	−	^***^
Total organ N (mg)		−	−	−	−	−	−	^***^
δ^15^N DM (‰)	−	−	−	−	−	−	−	^***^
δ^15^N WSF (‰)	−	−	−	−	−	−	−	^***^

*Nine durum wheat genotypes (genotype Forment de Artes was discarded due to late phenology) and three replicates per genotype (5 modern, i.e., semidwarf) cultivars for SI and RF conditions and four old (i.e., tall) cultivars only under RF conditions, 84 plots) were considered under rainfed N fertilized (RF+HN) and non-fertilized conditions (RF−LN) and supplemental irrigation N fertilized (SI+HN) and non-fertilized conditions (SI−LN). Sampling was performed 7 days after anthesis and the experiment was performed under field conditions during the 2011 crop season at the INIA's Experimental Station, Aranjuez, Spain. Mean values across plant tissues with different letters are significantly different according to the Tukey's honestly significant difference test (P < 0.05)*.

Level of significance: ns, not significant;

* P < 0.05;

** P < 0.01;

*** *P < 0.001*.

**Table 2:** **Genotype (G), water supply (W), nitrogen (N), and organ (O) effects (ANOVA) and mean values of nitrogen isotope composition (δ^15^N) in the water-soluble fraction (WSF) and dry matter (DM) of the flag leaf blade, peduncle, glumes, awns as well as in mature grains, nitrogen content (N content DM), total nitrogen content per organ (Total organ N), grain yield (GY), and total nitrogen of mature grains per spike (Grain N·spike^−1^)**.

	**Mean Values**				**ANOVA**			
	**SI+HN**	**SI−LN**	**WS+HN**	**WS−LN**	**G**	**W**	**N**	**O**
**FLAG LEAF**								
N content (%DM)	3.78a	3.40c	2.66b	2.08a	^**^	^***^	^***^	–
Total organ N (mg)	5.80c	6.53b	1.40a	1.46a	ns	^***^	ns	–
δ^15^N DM (‰)	3.70b	6.34c	−1.10a	4.52b	ns	^***^	^***^	–
δ^15^N WSF (‰)	3.55b	2.66b	−0.16a	1.74b	ns	^**^	ns	–
**PEDUNCLE**								
N content (%DM)	1.77c	1.59b	1.50b	1.33a	^***^	^***^	^**^	–
Total organ N (mg)	3.47c	3.13bc	2.57ab	2.23a	ns	^***^	ns	–
δ^15^N DM (‰)	4.07b	6.98c	−0.12a	4.29b	ns	^***^	^***^	–
δ^15^N WSF (‰)	3.57b	2.48b	−0.79a	1.46b	ns	^***^	ns	–
**GLUMES**								
N content (%DM)	1.63d	1.39b	1.53c	1.23a	^***^	^***^	^***^	–
Total organ N (mg)	1.13a	1.20a	1.28a	1.41a	ns	ns	ns	–
δ^15^N DM (‰)	3.22b	5.83c	−0.03a	4.12b	^*^	^***^	^***^	–
δ^15^N WSF (‰)	4.14b	3.25b	0.94a	2.17b	ns	^**^	ns	–
**AWNS**								
N content (%DM)	2.17c	1.88b	1.41a	1.24a	^***^	^***^	^***^	–
Total organ N (mg)	3.20b	2.93b	1.77a	1.90a	ns	^***^	ns	–
δ^15^N DM (‰)	3.09b	5.91c	0.35a	4.37b	^**^	^***^	^***^	–
δ^15^N WSF (‰)	5.1c	3.47b	0.71a	1.83ab	ns	^***^	ns	–
**GRAINS**								
N content (%DM)	2.48b	2.07a	2.39ab	2.21a	ns	ns	^**^	–
Grain N·spike^−1^	20.75b	21.94b	11.49a	13.92a	ns	^***^	^***^	–
δ^15^N DM (‰)	4.27b	6.6d	1.9a	5.53c	^**^	^***^	^***^	–
GY (Mg·ha^−1^)	4.37b	4.63b	1.68a	1.66a	ns	^**^	^***^	–
Nitrogen GY (Mg·ha^−1^)	0.12c	0.09b	0.04a	0.04a	^***^	^***^	^*^	–
N content (%DM)	–	–	–	–	–	–	–	^***^
Total organ N (mg)	–	–	–	–	–	–	–	^***^
δ^15^N DM (‰)	–	–	–	–	–	–	–	^***^
δ^15^N WSF (‰)	–	–	–	–	–	–	–	^***^

Ten durum wheat genotypes and three replicates per genotype (5 modern cultivars for SI and RF conditions and five old cultivars under RF conditions alone) were considered under rainfed N fertilized (RF+HN) and non-fertilized conditions (RF-LN) and supplemental irrigation N fertilized (SI+HN) and non-fertilized conditions (SI-LN). Sampling was performed 10 days after anthesis and the experiment was performed under field conditions during the 2012 crop season at the INIA's Experimental Station, Aranjuez, Spain. Mean values across plant tissues with different letters are significantly different according to the Tukey's honestly significant difference test (P < 0.05). Level of significance: ns, not significant;

* P < 0.05;

** P < 0.01;

*** *P < 0.001*.

In 2011 the average leaf chlorophyll content (SPAD) values across water and nitrogen conditions at the time of organ collection ranged between 52.9 and 52.6 for supplemental irrigation vs. rain-fed and between 55.8 and 49.8 for the fertilized and non-fertilized treatments, respectively (being only significant in fertilized conditions, data not shown). Regardless of the growing conditions, the total grain N content per spike (Grain N·spike^−1^) was much higher than the total N content of any of the different organs analyzed in 2011 and 2012 (Tables 1, 2). Moreover in 2011, irrespective of the organ analyzed and the water regime, the N content on a dry matter basis and the total organ N in fertilized conditions were higher than non-fertilized conditions (Table 1). Conversely, in 2012 and regardless of water regime, even though the N content on a dry matter basis was lower in non-fertilized (LN) than in fertilized (HN) conditions, the total organ N of all organs was higher in LN conditions with the exception of the peduncle (Table 2). At the same time, the flag leaf blade, peduncle, spike, roots, and awns together with mature grains exhibited higher total organ N content under support irrigation (SI) than rain-fed (RF) conditions (irrespective of nitrogen level) with the exception of the glumes (Tables 1, 2). In fact, organs that developed under SI conditions also showed larger dry weight than under RF conditions (Table S2). The organ with the highest total N content on a dry matter basis in 2011 was the spike (Tables 1), whereas in 2012 it was the flag leaf blade. Conversely, the glumes in 2012 showed the lowest N content on a dry matter basis (Table 2). With regards to genotypic differences, modern cultivars showed higher N content on a dry matter basis in the flag leaf, spike, peduncle, and awns than old cultivars, whereas total organ N was only higher in mature grains, the peduncle, and awns for modern cultivars compared to old cultivars (Table S3). Similarly, only N content showed a genotypic effect in all studied organs, whereas water supply, fertilization and organ showed an effect on almost all studied parameters (Tables 1, 2). Besides, the interaction of water supply, fertilization and organ was only significant for N content and for δ^15^N in WSF (see Table S1).

In 2011, the average nitrogen isotope composition in the dry matter (δ^15^N DM) within the four growing conditions was enriched in the grains (4.6%) and depleted in the roots (2.2%), whereas the spike and the flag leaf blade were between these values (3.8 and 3.5% for the flag leaf blade and the spike, respectively; Table 1). Similarly, in 2012 the δ^15^N DM of all organs studied (flag leaf blade, peduncle, glumes, and awns) was depleted compared to mature grains (4.5%) (Table 2). However, during both seasons the δ^15^N was enriched in all organs under non-fertilized conditions (SI-LN and RF-LN) compared to fertilized conditions (SI+HN and RF+HN). In particular, the δ^15^N in all organs under LN conditions was enriched when associated with SI plots (SI-LN) compared with RF plots (RF-LN) for both growing seasons (Tables 1, 2). Similarly, under fertilized conditions, the δ^15^N in all organs was enriched when associated with SI plots (SI+HN) compared to RF plots (RF+HN). With regard to the WSF, δ^15^N was generally lower (depleted) than in the DM but the trends across water and fertilization regimes and genotypes were similar (Tables 1, 2). However, modern cultivars showed enriched δ^15^N only in mature grains compared to old cultivars (Table S3).

### Potential and relative organ N contribution to grain N

**Figure 1:**
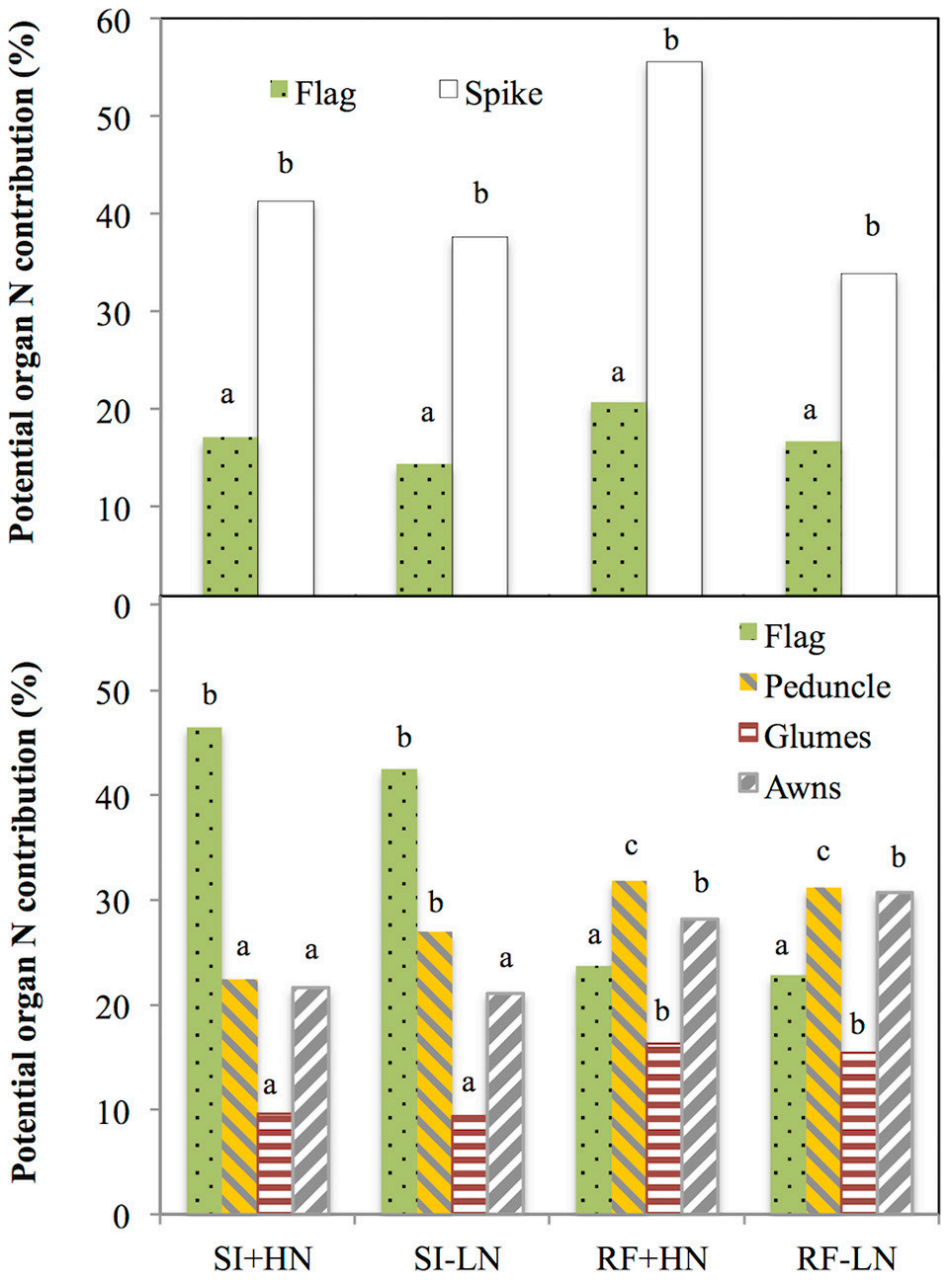
**Potential N contribution of the flag leaf blade, peduncle, spike, glumes, and awns to the nitrogen accumulated in the grains**. Values were calculated as the product of nitrogen content (N content) of the different organs multiplied by their respective dry weight and standardized by the total nitrogen content of mature grains per spike (Grain N·spike^−1^). For the total spike N calculation, total grain N of developing grains was subtracted from the calculation (see Section Materials and Methods). Ten durum wheat genotypes (genotype Forment de Artes was discarded due to late phenology in 2011) and three replicates per genotype (totalling 84 plots in 2011 and 90 plots in 2012) were considered under rainfed N fertilized (RF+HN) and non-fertilized conditions (RF−LN) and supplemental irrigation N fertilized (SI+HN) and non-fertilized conditions (SI−LN). Sampling was performed 7 and 10 days after anthesis (2011 and 2012, respectively) and the experiment was performed under field conditions at the INIA's Experimental Station, Aranjuez, Spain during the 2011 (upper panel) and 2012 (lower panel) growing seasons. Mean values across organs and different growing conditions with different letter are significantly different according to the Tukey's honestly significant difference test (*P* < 0.05).

The potential contribution of the entire ear as a source of N to the grain nitrogen (grain N) was on average (across all growing conditions) almost double (42%) to that of the flag leaf blade (17%) in 2011 (Figure 1, upper panel), whereas in 2012 the potential N contribution of the flag leaf blade (33%) to grain N was higher than in the main individual tissues of the spike such as the glumes (13%) or awns (13%) as well as higher than in the peduncle (28%) (Figure 1, lower panel). Further, the potential N contribution (i.e., compared with the other plant parts studied) of the flag leaf as a source of N to grain N increased to a 46% with better growing conditions (SI+HN) in 2012 (Figure 1, lower panel), whereas the opposite trend occurred for the entire spike (50% in 2011), peduncle (31% in 2012) and glumes and awns (23% in 2012). Indeed, the sum of the potential N contribution of awns and glumes to the total N accumulated in the grains (46%) under RF was comparable to that of the flag leaf blade (46%) under SI conditions (Figure 1, lower panel). Moreover, the ratio between the weights of all analyzed organs under RF divided by the weight under SI conditions (Table S2) was lower in the flag leaf (0.42) than other spike organs such as glumes (0.88) and awns (0.77). Furthermore, regardless of the growing conditions, old cultivars showed higher potential N content in the whole spike, its specific parts (glumes and awns) and the organs below the spike (peduncle and flag leaf) than in the modern cultivars (Figure S1). However, no genotypic effect was observed on the potential N content of the different organs (Table S4).

**Figure 2:**
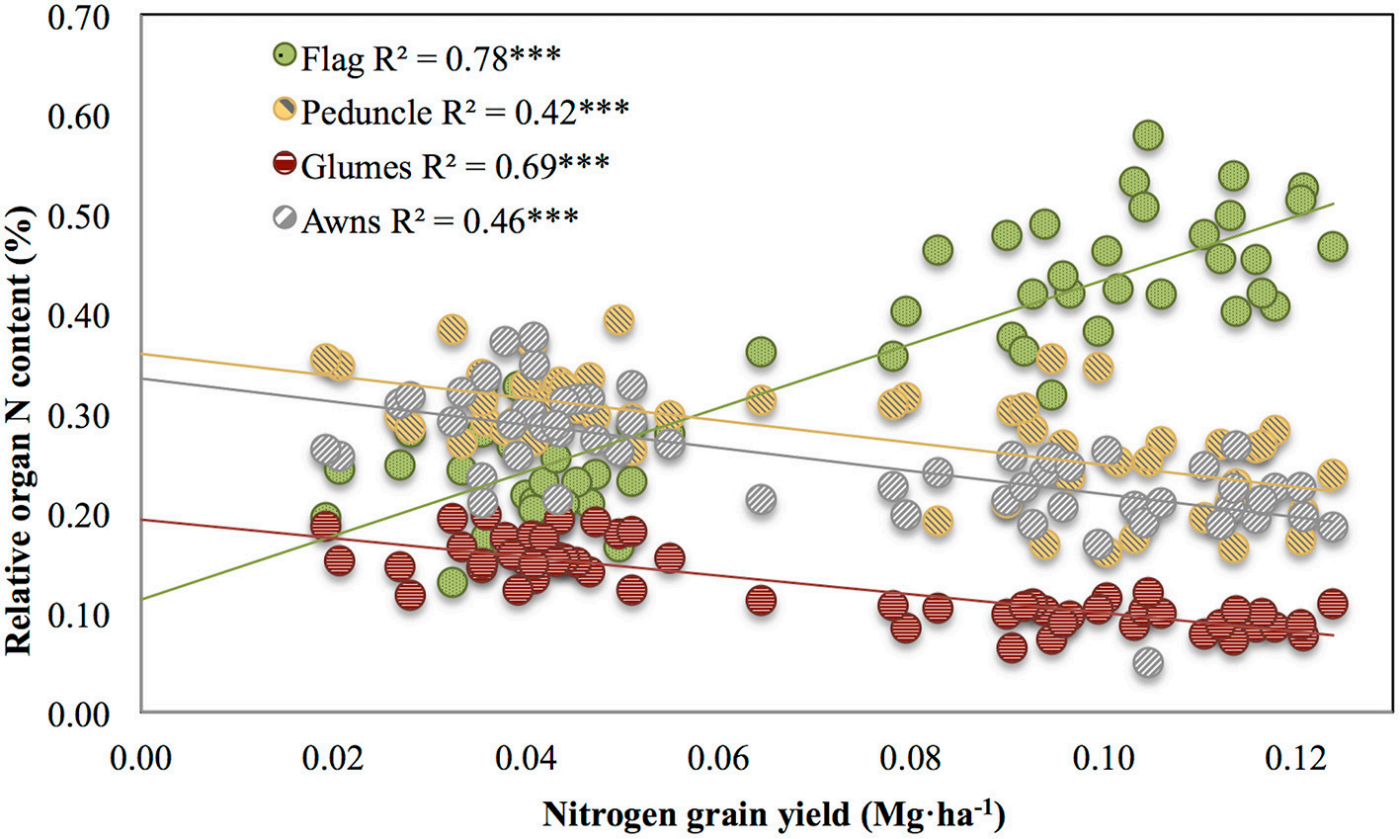
**Pearson correlations between the nitrogen grain yield and the relative organ N content of the flag leaf blade, peduncle, glumes, and awns across a wide range of environmental conditions**. The relative contribution of the different organs was calculated as the value of the potential organ N contribution for the specific organ divided by the sum of the potential organ N contribution of all the organs studied (flag leaf blade, peduncle, glumes, and awns). Sampling was performed 10 days after anthesis. Ten durum wheat genotypes and three replicates per genotype (totalling 90 plots) were considered, including the four growing conditions (RF+HN, RF−LN, SI+HN, and SI−LN) tested at the INIA's Experimental Station, Aranjuez, Spain in 2012. Levels of significance: ***P < 0.001.

Correlations across growing conditions between nitrogen grain yield and the relative N content of glumes (*R*^2^ = 0.69, *P* < 0.001), awns (*R*^2^ = 0.46, *P* < 0.001), and peduncle (*R*^2^ = 0.42, *P* < 0.001) were negative (Figure 2), whereas the same category of relationships in the flag leaf were positive (*R*^2^ = 0.78, *P* < 0.001). Thus, whereas the relative N content of the flag leaf with regard to the nitrogen GY increased with better growing conditions (and thus higher nitrogen grain yield), the relative N contribution in the glumes, awns, and peduncle increased under more stressed conditions (low nitrogen grain yield; Figure 2).

**Figure 3:**
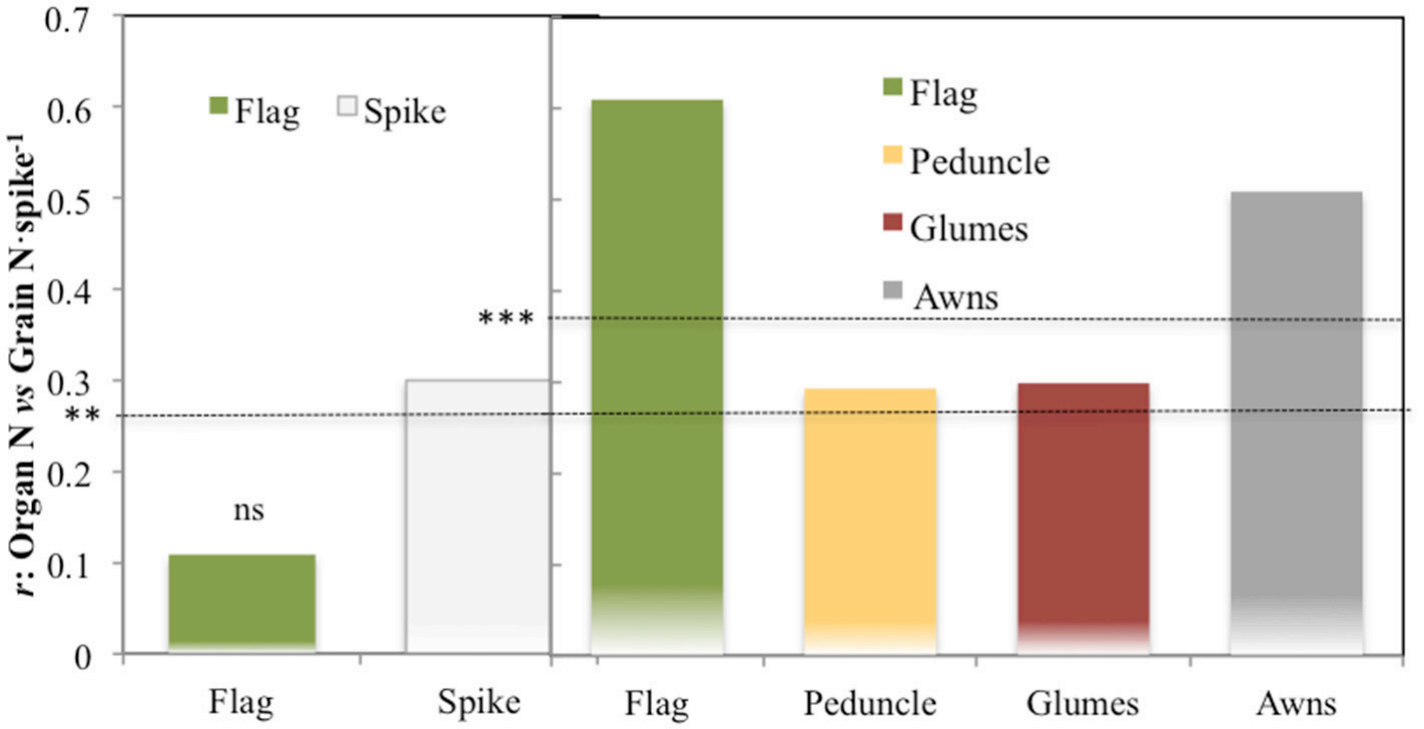
**Correlation coefficients of the relationships of the total N content per organ against the total N accumulated in the grains of the spike**. The different plant organs assayed were: the flag leaf blade and spike (2011) and the flag leaf blade, peduncle, glumes, and awns (2012). For the calculation, five modern cultivars for SI and RF conditions and five old cultivars under RF conditions alone were considered, with 84 plots in 2011 (as genotype Forment de Artes was discarded due to late phenology in 2011) and 90 in 2012 including the four growing conditions (RF+HN, RF−LN, SI+HN and SI−LN) tested in the INIA's Experimental Station in Aranjuez, Spain in 2011 and 2012. Growing conditions as detailed in the legend of Figure 1. Levels of significance: ns, not significant; **P < 0.01; ***P < 0.001.

The correlations of total N content per organ against the total grain N content per spike across growing conditions supported a greater role for the spike as a whole than the flag leaf blade, at least under the low yielding conditions of 2011. Thus the total N in the spike was better related to total grain N content per spike than the total N in the flag leaf (Figure 3, left panel). However, for the 2012 season, correlations of total organ N content, and total grain N content per spike were positive and significant for all the plant parts studied, but those of the flag leaf blade and also the awns were slightly higher (*P* < 0.001) than those of the peduncle and the glumes (Figure 3, right panel) in 2012. These results suggest an increase in the role of the flag leaf blade as a source of N to the grains as growing conditions improved (Figure 3, right panel).

The relationships between HI and the potential N contribution of the different organs across all genotypes and growing conditions were studied (**Table 4**). Except for the flag leaf in 2012 a negative correlation between the potential N contribution of a given organ and HI was observed, with old cultivars exhibiting, in general, higher ear contributions and lower HIs than the modern cultivars.

### Fractionation nitrogen isotope composition

**Figure 4:**
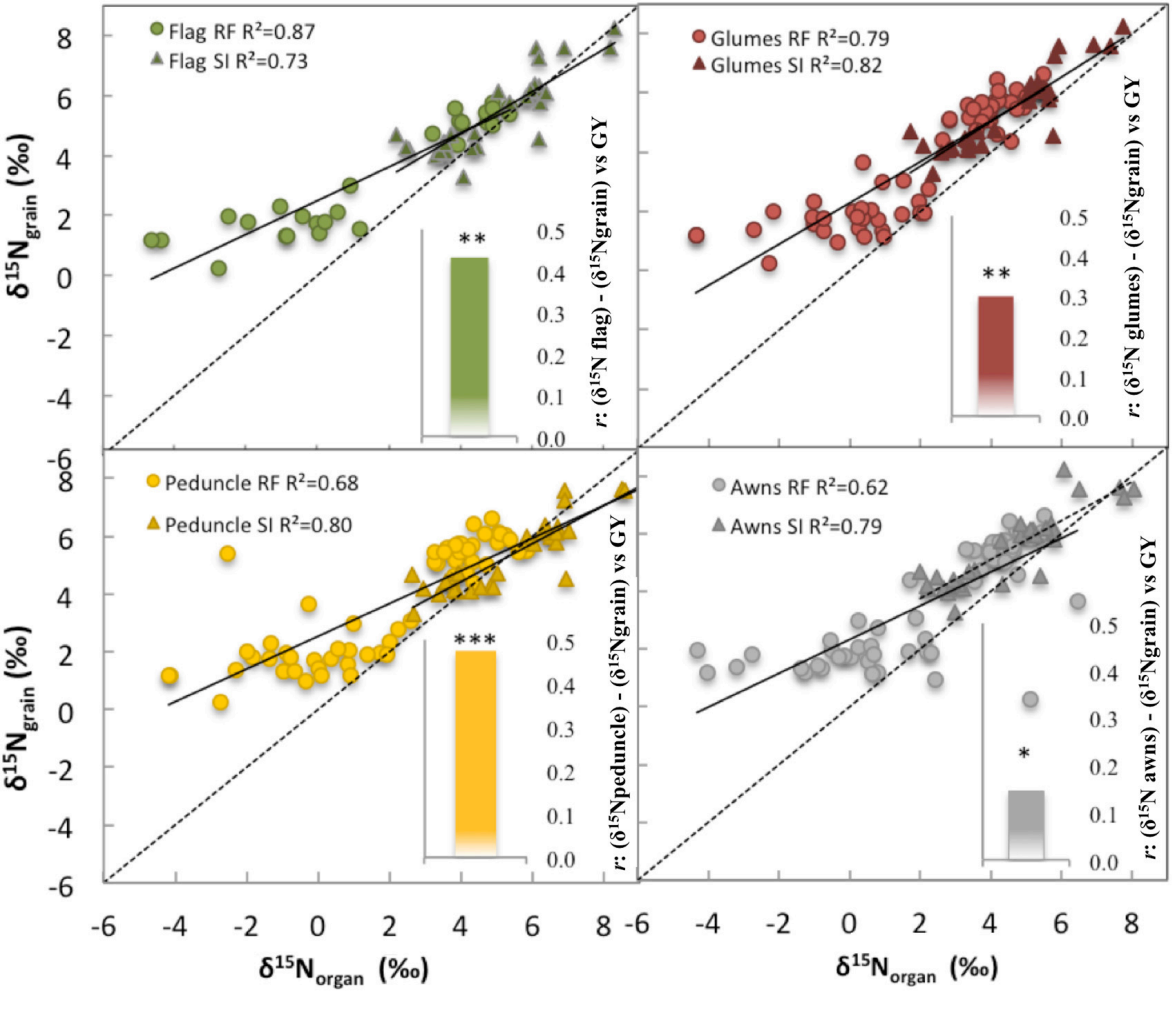
**Linear regression of the relationship between the nitrogen isotope composition (δ^15^N) in the mature grains (δ^15^Ngrain) against the δ^15^N in the dry matter of the flag leaf blade, peduncle, glumes, and awns**. Figure inset: nitrogen isotope fractionation during translocation to the grains from source organs (flag leaf blade, peduncle, glumes, and awns) to grains in wheat (see figure inset), are displayed as the relationship between the difference in δ^15^N_organs_ minus δ^15^N_grain_ (fractionation) vs. grain yield (GY). Ten durum wheat genotypes and three replicates per genotype (90 plots in 2012) were considered including the four growing conditions (RF+HN, RF−LN, SI+HN, and SI−LN) tested at the INIA's Experimental Station in Aranjuez, Spain in 2011 (upper panel) and 2012 (lower panel). Sampling was performed 7 and 10 days after anthesis in 2011 and 2012, respectively. Levels of significance: ns, not significant; *P < 0.05; **P < 0.01; ***P < 0.001.

**Figure 5:**
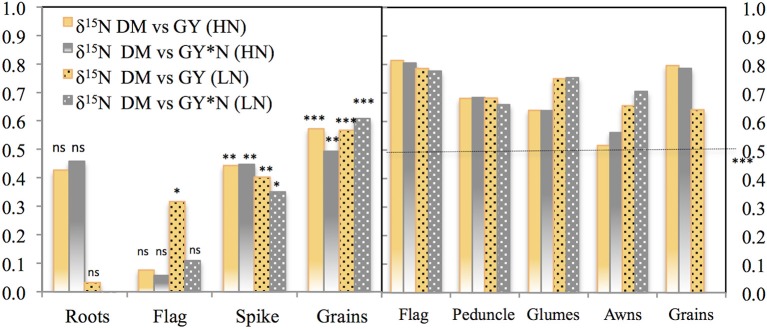
Pearson correlations of the nitrogen grain yield (GY*Ncontent grain) and grain yield (GY) against the nitrogen isotope composition (δ^15^N) in the dry matter (DM) of the roots, flag leaf blade, peduncle, spike, glumes, and awns. Ten durum wheat genotypes and three replicates per genotype (84 plot in 2011, left panel; and 90 plots in 2012, right panel) were considered including the four growing conditions (RF+HN, RF−LN, SI+HN, and SI−LN) tested at the INIA's Experimental Station, Aranjuez, Spain in 2011 (left panel) and 2012 (right panel). Sampling was performed 7 and 10 days after anthesis in 2011 and 2012, respectively. Levels of significance: ns, not significant; *P < 0.05; **P < 0.01; ***P < 0.001.

Strong linear correlations (including all growing conditions) were observed between the δ^15^N in the mature grains and the δ^15^N in the dry matter of all studied plant organs and growing seasons (*P* < 0.001; Table 3, Figure 4). Nevertheless, the strongest correlation against the δ^15^N of the grains was achieved by the flag leaf blade (*r* = 0.96 *P* < 0.001; Table 3, Figure 4, high-left panel) followed by the entire spike (*r* = 0.92 *P* < 0.001) and the glumes (*r* = 0.91 *P* < 0.001). In addition, in the relationship between the δ^15^N in the grains and the corresponding values within each organ represented in Figure 4, the higher the δ^15^N in the grains, the more similar the δ^15^N values of the different organs and the δ^15^N in the grains (Figure 4). Conversely, the more depleted the δ^15^N in the grains the more different (further below) the δ^15^N values in the different organs and the δ^15^N in the grains in 2011 (Figure S2) and 2012 (Figure 4). Specifically, the δ^15^N in the flag leaf blade showed the most different values (further below) compared with the δ^15^N of the grains, especially under RF conditions. Conversely the δ^15^N in the glumes and awns showed constant (i.e., regardless of the δ^15^N of the grains) equidistant values away from the δ^15^N in the grains (Figure 4, left-upper and lower panel). Nevertheless, nitrogen fractionation from source organs to the sink (assumed to be the difference between δ^15^N_organs_ minus δ^15^N_grains_) increased with improvements in growing conditions as observed by the increases in the difference between the δ^15^N_organs_ minus δ^15^N_grains_ associated with an increase in the GY (Figure 4, see figure inset). In particular, the flag leaf showed the highest nitrogen fractionation associated with the increase in GY, whereas the awns showed the lowest nitrogen fractionation associated with GY (Figure 4, right lower panel, see figure inset). In addition, in 2011 the δ^15^N in the flag leaf was positively and strongly related to stomatal conductance (*r* = 0.75; *P* < 0.001) under RF conditions (Figure S3). Moreover, δ^15^N in the different organs was positively related to GY and nitrogen grain yield in 2011 and 2012 (Figure 5). The organ where δ^15^N was best related to GY was the spike (*P* < 0.001) in 2011 and the flag leaf blade (*P* < 0.001) in 2012 (Figure 5, right and left panels, respectively).

**Table 3:** **Linear regression of the relationship between the nitrogen isotope composition (δ^15^N) in the mature grains (δ^15^N grain DM) against the δ^15^N in the dry matter (DM) and water-soluble fraction (WSF) of the flag leaf blade, spike and roots (2011) and flag leaf blade, peduncle, glumes and awns (2012)**.

	***vs*. δ^15^N grain DM**									
	**SI+HN**		**SI−LN**		**WS+HN**		**WS−LN**		**Global**	
**2011**										
δ^15^N DM	−0.136	ns	0.545	ns	−0.086	ns	−0.559	ns	**0.801**	^***^
**Flag Leaf**										
δ^15^N DM	**0.810**	^***^	**0.636**	^*^	**0.566**	^**^	0.242	ns	**0.957**	^***^
δ^15^N WSF	**0.807**	^**^	**0.686**	^**^	0.323	ns	0.083	ns	**0.948**	^***^
**Spike**										
δ^15^N DM	0.457	ns	0.247	ns	**0.431**	^*^	0.204	ns	**0.920**	^***^
δ^15^N WSF	**0.510**	^*^	**0.704**	^*^	**0.566**	^**^	−0.019	ns	**0.935**	^***^
**2012**										
**Flag Leaf**										
δ^15^N DM	0.043	ns	**0.761**	^**^	**0.533**	^*^	**0.609**	^*^	**0.932**	^***^
δ^15^N WSF	0.198	ns	**0.803**	^***^	0.002	ns	−**0.562**	^**^	**0.397**	^***^
**Peduncle**										
δ^15^N DM	0.364	ns	**0.857**	^***^	**0.610**	^***^	0.212	ns	**0.869**	^***^
δ^15^N WSF	0.156	ns	0.441	ns	−0.034	ns	−**0.562**	^**^	**0.453**	^***^
**Glumes**										
δ^15^N DM	0.377	ns	**0.860**	^***^	**0.541**	^**^	**0.508**	^**^	**0.910**	^***^
δ^15^N WSF	0.112	ns	0.157	ns	−0.076	ns	−**0.431**	^*^	**0.313**	^**^
**Awns**										
δ^15^N DM	0.165	ns	**0.747**	^**^	0.263	ns	**0.585**	^**^	**0.836**	^***^
δ^15^N WSF	−0.059	ns	0.434	ns	−0.181	ns	−**0.518**	^**^	**0.293**	^**^

Ten durum wheat genotypes and three replicates per genotype (five modern cultivars for SI and RF conditions and five old cultivars only under RF conditions, 84 plots in 2011, and 90 in 2012 as genotype Forment de Artes was discarded due to late phenology in 2011) were considered under rainfed N fertilized (RF+HN) and non-fertilized conditions (RF-LN) and supplemental irrigation N fertilized (SI+HN) and non-fertilized conditions (SI-LN). Sampling was performed 7 and 10 days after anthesis in 2011 and 2012, respectively and the experiment was performed under field conditions at the INIA's Experimental Station, Aranjuez, Spain, during two consecutive seasons (2011 and 2012). The significant correlations are marked in bold. Levels of significance: ns, not significant;

* P < 0.05;

** P < 0.01;

*** *P < 0.001*.

## Discussion

Grain yield (GY) during 2011 was lower than in 2012, and was within the range of GY previously reported under very severe drought stress conditions in the Mediterranean basin (Araus et al., 1998; Oweis et al., 2000). In addition, the interaction of nitrogen and water showed an effect on GY (Table S1). During both years support irrigation had a positive effect on GY, whereas, nitrogen fertilization did not have any positive effect on GY except for SI in 2011, and otherwise tended to have the opposite effect (Tables 1, 2). N fertilization could have caused haying off, thus decreasing GY, which might have been triggered by a terminal stress during reproductive stage (Araus et al., 2013).

### Effect of water and N fertilization in nitrogen isotope composition

RF conditions caused a decrease in organ δ^15^N (flag leaf, peduncle, glumes, awns, and grains) compared with the irrigated trial. Likewise, a decrease in δ^15^N under stress conditions has been previously reported in shoots (Yousfi et al., 2012) and mature grains of durum wheat (Araus et al., 2013) and bread wheat (Robinson, 2000). The increase in δ^15^N in response to SI may be the consequence of labile nitrate derived from chemical fertilizers (urea in our case) with a depleted (near to zero) δ^15^N (Bateman and Kelly, 2007) leaching out of the root zone (Hawkesford, 2014) to lower subsoil layers (Raimanová and Haberle, 2010). Alternatively, the increase in plant biomass due to irrigation may lead to exhaustion of the nitrogen fertilizer, therefore causing the crop to rely on natural sources of soil N, which are characterized by higher δ^15^N values (Serret et al., 2008). Therefore, apart from chemical fertilizers, nitrogen pools derived from the mineralization of soil organic matter and with an δ^15^N enriched signature (Raimanová and Haberle, 2010) may become available to the plant (Evans and Belnap, 1999).

### Nitrogen isotope composition and grain yield

Linear correlations of organ δ^15^N with GY and nitrogen GY were strong and positive for both growing seasons (Figure 5), as has also been observed in the past with maize (Coque et al., 2006) and durum wheat (Yousfi et al., 2009). Such positive relationships between δ^15^N and GY could be related to some extent with stomatal conductance (Farquhar et al., 1980; Araus et al., 2013). Accordingly, stomatal conductance under RF conditions was positively related to δ^15^N in the flag leaf blade in 2011 (*r* = 0.75, *P* < 0.001, Figure S3). Thus, low stomatal conductance may reduce losses of ammonia and nitrous oxides which would reduce the enrichment of δ^15^N (Farquhar et al., 1980; Smart and Bloom, 2001). In fact, linear regression between δ^15^N in the mature grains and δ^15^N in the dry matter of the different organs (Figure 4) supports this finding. Thus, the enricher was the δ^15^N in the grains; the more similar were the δ^15^N values of the different organs to the δ^15^N in the grains (slope 1:1 of the relationships between δ^15^N of grains and organs, respectively). Thus, taking into account that for a given nitrogen fertilization value higher values of δ^15^N represented better growing conditions (observed by the positive correlation between GY and δ^15^N), enrichment of δ^15^N due to either N volatilization or leakage and/or exhaustion of chemical fertilizer (with a lower δ^15^N) probably occurred; as a consequence values of organ δ^15^N became closer to the δ^15^N in the grains (Raimanová and Haberle, 2010). Conversely, different values of δ^15^N in the grains in comparison to the δ^15^N of the different organs suggest that reduction in stomatal conductance in response to RF conditions prevented enrichment in the δ^15^N of the grains. In addition, the flag leaf blade showed the highest nitrogen fractionation associated with the increase in GY compared with the awns, glumes, and peduncle (Figure 4, see figure inset). This finding suggests that losses of ^14^N due to volatilization were higher in the flag leaf due in part to higher stomata density (and thus conductance and transpiration) in the flag leaf (which is an amphystomatic organ) than in the awns (Tambussi et al., 2005; Li et al., 2006). In addition, the δ^15^N in the roots also showed a similar trend, suggesting that N losses through volatilization or exudation might also be occurring in the roots (Johansson et al., 2009; Figure S1). However, the δ^15^N in field experiments should be interpreted with caution as miscellaneous biotic and abiotic factors can affect the natural abundance and discrimination of δ^15^N in the soil-plant system (Hogberg, 1997; Robinson et al., 1998; Evans, 2001; Robinson, 2001; Cernusak et al., 2009; Yousfi et al., 2012). In fact, in our study fractionation of δ^15^N was present (Figure 4) because the δ^15^N of the grains was increased in comparison to the values in the individual photosynthetic organs (Figure 4). Linear correlations of GY and nitrogen GY against the organ δ^15^N in the WSF were positive for both growing seasons but weaker (data not shown) compared with the correlation against organ δ^15^N in the dry matter. Such weaker correlations in the organ δ^15^N WSF might be related to the fact that the WSF is protein-free because enzymatic N is removed from the WSF and it only contains free amines (Cabrera-Bosquet et al., 2011).

### Relative and potential organ contribution to grain nitrogen

The relative (i.e., compared with the other plant parts studied) and potential (i.e., relative value with regard to the total N accumulated in the grains of a spike) contributions of the flag leaf as a source of N for grain nitrogen (grain N) increased as growing conditions improved at least during the second year, whereas the opposite occurred for the peduncle, glumes, awns, and entire spike (Figure 1). The flag leaf blade has been reported as the main nitrogen exporter to the grains in bread wheat (Simpson et al., 1983). In spite of the high contribution traditionally assigned to the flag leaf blade as a source of N to the growing grains (Evans, 1983; Araus and Tapia, 1987), in our study, the potential contribution of the flag leaf blade shortly after anthesis was <50% (regardless of the growing conditions; Figure 1). Leaf N remobilization to grain N in rice, wheat or maize has been observed to vary from 50 to 90% (Masclaux et al., 2001). Contrastingly, Simpson et al. (1983) reported that less than half of the nitrogen retranslocated from the leaves arrives directly in the grain, whereas the rest is mostly translocated to the roots. However, most of the nitrogen translocated to the roots is further retranslocated to the shoots via xylem sap (Simpson et al., 1983) where it supplies transpirative organs such as the glumes, leaves, and stem (Simpson et al., 1983). However, N directly exported from the roots to non-transpirative organs such as the grains may be a minor player because grains may receive only 1% of the nitrogen exported from the roots (Simpson et al., 1983). Additionally, the relatively low potential N contribution of the flag leaf blade to grain N may also indicate that aside from other parts of this leaf (such as the sheath), other organs of the plant contribute to the N accumulated in the grains. Leaves below the flag leaf were not considered in this study as their potential contribution to grain N is reported to be minor Del Pozo et al. (2007). Therefore we only considered the upper part of the plant, including the peduncle and the spike (Figure 1, upper panel and lower panel). This view is supported by the strong linear correlations (including all growing conditions) between the δ^15^N in the mature grains and the δ^15^N of the dry matter of these plant organs (*P* < 0.001; Table 3). In spite of this, the strongest correlation against δ^15^N in the mature grains was achieved by the flag leaf blade followed by the glumes and the entire spike (Table 3). Additionally, the correlations of the total N content per organ against the total grain N content per spike also support a slightly greater role for the flag leaf than the other plant parts (Figure 3, right panel). Besides, the experiment conducted under more severe stress in 2011 (growth cycle with low GY) supports the important role of the spike to grain N, since N content in the entire spike was better related to Grain N·spike^−1^ in comparison to the flag leaf (Figure 3, left panel). Furthermore, the role of the flag leaf sheath supplying N to the growing grains should be not neglected as a potential source of N (Araus and Tapia, 1987).

Therefore, apart from the potential contribution of the flag leaf (and eventually other vegetative organs), the role of the ear as a supplier of N should be taken into account, as total N allocation in the ear has been observed to be higher than in the flag leaf blade in durum wheat in field chambers (Vicente et al., 2015a). Indeed, accumulation of nitrogen in the grains is closely dependent on N mobilization originating from the glumes (23% of N contribution to the grain) in the absence of an exogenous supply of nitrogen (Simpson et al., 1983). Similarly, in our study the potential and relative N contribution of the awns and glumes as well as the peduncle with respect to the flag leaf blade increased under water stress and non-fertilized conditions (Figures 1, 2, respectively). In fact, the sum of the potential N contribution of awns and glumes under RF conditions was comparable to that of the flag leaf blade under SI conditions (Figure 1). In a recent study performed in wheat grown hydroponically, the total N accumulated in the flag leaf blade was comparatively lower than the amount accumulated in the remaining upper parts of the plant (Vicente et al., 2015b). The lower potential N content of the flag leaf blade under RF conditions could be related to organ size but was not concomitant with leaf senescence (as chlorophyll content was similar under RF and SI conditions at the moment of sampling, data not shown). Thus, the ratio between the weight of all analyzed organs under RF divided by the SI conditions (Table S2) was lower in the flag leaf (0.42) than other spike organs such as glumes (0.88) and awns (0.77). These results suggest that the leaf lamina was smaller under RF, whereas the spike size (entire spike, awns, and glumes) was not reduced under RF conditions (Table S2). On the other hand, growing conditions may also affect the efficiency of N transfer from source organs to the grains (Masclaux-Daubresse et al., 2010). For example, part of the N accumulated in the flag leaf and other leaves may be exported back to the roots, particularly under stress (drought, low fertility) to promote root development mainly in vegetative stages (Palta and Gregory, 1997) and to a lesser extent during grain filling (Jensen, 1994; Swinnen et al., 1994). Conversely, during rapid grain filling glumes may only retranslocate N to the grains, thus increasing the role of N under water stress conditions (Waters et al., 1980). In addition, the potential N contribution of the ear, peduncle and glumes was higher in old than modern cultivars (Figure S2). Such differences might be related, at least in part, to the larger harvest index (HI) in modern cultivars than old cultivars (Reynolds et al., 2009; Foulkes et al., 2011; Sanchez-Bragado et al., 2014a) due to the introduction of *dwarfism alleles* (Maydup et al., 2012). The negative correlations between the potential organ nitrogen contribution and HI (Table 4) suggest that old cultivars may provide a greater contribution of N to the grains in relative terms, especially from the ear due to a sink driven phenomenon. In a semidwarf (i.e., modern) genotype the absolute amount of N accumulated in the peduncle may be lower than in a tall (i.e., old) cultivar, although not significant differences were observed (Table S1). Conversely, larger spike in the modern cultivars decrease the surface (where the photosynthetic tissues accumulating N are placed) to volume (where grain N is accumulated) ratio in comparison to spikes in the old cultivars. That is, the spike surface (assumed to be the photosynthetic tissues of the spike such as the glumes and awns) decreases relative to the spike volume (assumed to be the grains). Thus, in modern cultivars a lower relation between spike surface and volume may have decreased the potential of the ear to provide nitrogen to the grains compared to old cultivars. Consequently, these results suggest that among the set of genotypes studied, potential spike N content is not only dependent on growing conditions but also on genotype (sink strength and plant height).

**Table 4:** **Linear regression of the relationship between the HI and Potential N contribution of the flag leaf blade, peduncle, spike, glumes, and awns to the nitrogen accumulated in the grains**.

	***vs*. HI**		
	**Potential organ N contribution**	***r***	**Sig**.
2011	Flag	−0.353	0.001
	Spike	−0.315	0.004
2012	Flag	0.227	0.083
	Peduncle	−0.382	0.000
	Glumes	−0.539	0.000
	Awns	−0.357	0.001

*Values were calculated as the product of nitrogen content (N content) of the different organs multiplied by their respective dry weight and standardized by the total nitrogen content of mature grains per spike (Grain N·spike^−1^). For the total spike N calculation, total grain N of developing grains was subtracted from the calculation (see Section Materials and Methods). Ten durum wheat genotypes and three replicates per genotype (five modern cultivars for SI and RF conditions and five old cultivars under RF conditions alone, 84 plots in 2011 and 90 in 2012 as the genotype Forment de Artes was discarded due to late phenology in 2011) were considered combining all growing conditions under rainfed N fertilized (RF+HN) and non-fertilized conditions (RF-LN) and supplemental irrigation N fertilized (SI+HN) and non-fertilized conditions (SI-LN). Sampling was performed 7 and 10 days after anthesis (2011 and 2012, respectively) and the experiment was performed under field conditions at the INIA's Experimental Station, Aranjuez, Spain during the 2011 and 2012*.

Summarizing, the significant correlations between the total N content of the different plant organs studied (flag leaf blade, peduncle, glumes, and awns) against the grain N per spike, suggest that all these organs can potentially export a proportion of their N to the grains. This view was supported by the strong linear correlations (including all growing conditions) between the δ^15^N in the mature grains and the δ^15^N in the dry matter of all studied plant organs. Moreover, the large amount of N accumulated in the whole grains of the spike, together with the relatively low amount of N available in the different organs supports the concept that N imported into the grains cannot be sustained by one organ alone; rather, different organs may simultaneously export nitrogen to the grains. In spite of that, the role of the flag leaf blade as a potential supplier of N to grains increased, in comparison to other upper parts of the plant, under improved growing conditions (and thus higher GY) as well as in the modern (semidwarf) cultivars compared to the old cultivars. In contrast, the relative importance of the ear and peduncle increased under water stress conditions (low GY) or in the old genotypes compared to the new genotypes. Such findings indicate that other than the flag leaf (and eventually other vegetative organs), the role of the ear as a supplier of N should be taken into account even though growing conditions may affect the relative potential contribution of the different plant parts. Thus, the potential ear N content could be a positive trait for plant phenotyping, especially under water limiting and/or low fertility conditions. The total N content of the spike at early grain filling should be considered a trait amenable in crop management (e.g., precision agriculture) as well as for breeding (phenotyping). Nevertheless, the challenge is to find high throughput monitoring techniques for this trait. Besides the specific results achieved, the objective of this study was to test methodologies to asses the potential N contribution of different organs to developing grains. It does not pretend to in detail estimate the integrated in time contributions of different organs to grain N throughout grain filling, but to provide a comparative view across organs potentially useful as an affordable phenotyping tool.

## Author contributions

RS and JA conceived designed the study; RS and MS carried out the field measurements; RS conducted laboratory work, RS and JA analyzed the data; RS and JA interpreted the results; RS took the principal role in writing the manuscript under supervision of JL. All authors have contributed to the revision of the manuscript.

### Conflict of interest statement

The authors declare that the research was conducted in the absence of any commercial or financial relationships that could be construed as a potential conflict of interest.
