# Supplementary material for: The Nitrogen Contribution of Different Plant Parts to Wheat Grains: Exploring Genotype, Water, and Nitrogen Effects
Source: Front Plant Sci. 2017 Jan 9;7:1986. doi: 10.3389/fpls.2016.01986 (PMC5220073; doi:10.3389/fpls.2016.01986)
Supplement: Supplementary file 1 [file DataSheet1.pdf]

SUPPLEMENTAL INFORMATION

The nitrogen contribution of different plant parts to wheat grains: exploring genotype, water and nitrogen effects

Sanchez-Bragado R, Serret M[1], Araus JL

Interactions	Dependent variable	Level of significance
Water * Nitrogen	N content (%DM)	0.112
	Total organ N (mg)	0.904
	$\delta^{15}N$ DM (‰)	0.001
	$\delta^{15}N$ WSF (‰)	0.000
Water * Organ	N content (%DM)	0.000
	Total organ N (mg)	0.014
	$\delta^{15}N$ DM (‰)	0.000
	$\delta^{15}N$ WSF (‰)	0.000
Nitrogen * Organ	N content (%DM)	0.000
	Total organ N (mg)	0.000
	$\delta^{15}N$ DM (‰)	0.001
	$\delta^{15}N$ WSF (‰)	0.000
Water * Nitrogen * Organ	N content (%DM)	0.009
	Total organ N (mg)	0.667
	$\delta^{15}N$ DM (‰)	0.068
	$\delta^{15}N$ WSF (‰)	0.023
Water * Nitrogen	2010 GY ($Mg\cdot ha^{-1}$)	0.000
	2011 GY ($Mg\cdot ha^{-1}$)	0.003

Table S1. Interaction of water, nitrogen and the organ effect (ANOVA) on the nitrogen isotope composition ($\delta^{15}N$) in the water-soluble fraction (WSF) and dry matter (DM) of all organs, nitrogen content (N content), total nitrogen content per organ (Total organ N). Ten durum wheat genotypes (genotype Forment de Artes was discarded due to late phenology in 2011) and three replicates per genotype were considered by combining all growing conditions totalling 84 plots in 2011 and 90 in 2012. The experiment was performed under field conditions during the crop seasons, 2011 and 2012 at the INIA's Experimental Station, Aranjuez, Spain. The associated percentage of the sum of squares and probabilities calculated through the analysis of variance (ANOVA) is shown for water (W), nitrogen (N) and organ (O) and the interaction between them. Levels of significance are as follows: ns, not significant; *$P < 0.05$; **$P < 0.01$; ***$P < 0.001$.

		Organ dry weigth RF/ Organ dry weight SI
2011	**Flag leaf**	1.05
	**Spike**	1.05
2012	**Flag**	0.42
	**Peduncle**	1.15
	**Glumes**	0.88
	**Awns**	0.77

Table S2. Ratio between dry weight (g) of the spike, flag leaf, peduncle, glumes and awns under rain-fed (RF) divided by supplemental irrigation (SI) conditions. Nine durum wheat genotypes and three replicates per genotype were considered combining rainfed fertilized and non-fertilized conditions (RF) and supplemental irrigation under fertilized and non-fertilized conditions (SI). The experiment was performed under field conditions during the crop seasons 2011 and 2012 at the INIA's Experimental Station, Aranjuez, Spain.

Table S3. Mean values of nitrogen isotope composition ($\delta^{15}N$) in the water-soluble fraction (WSF) and dry matter (DM) of the flag leaf blade, peduncle, spike, glumes and awns as well as in mature kernels (grains), nitrogen content (N content), total nitrogen content per organ (Total organ N), grain yield (GY) and total nitrogen content of mature kernels per spike (Grain N·spike^{-1}). Five modern cultivars (60 plots in 2011 and 2011) and five old genotypes (24 plots in 2011, 30 plots in 2012) of durum wheat genotypes (old genotypes under SI conditions were discarded in 2011 and 2012) and three replicates per genotype were considered combining all growing conditions. The experiment was performed under field conditions during the crop seasons 2011 and 2012 at the INIA's Experimental Station, Aranjuez, Spain. Mean values across plant tissues with different letters are significantly different according to the Tukey's honestly significant difference test (P<0.05).

		N content DM (%)		Total organ N (mg)		$\delta^{15}N$ DM (‰)		$\delta^{15}N$ WSF (‰)		GY (Mg·ha^{-1})		Nitrogen GY (Mg·ha^{-1})	
		Old	Modern	Old	Modern	Old	Modern	Old	Modern	Old	Modern	Old	Modern
**2011**	Flag leaf	3.2^{a}	3.4^{a}	3.8^{a}	3.9^{a}	3.6^{a}	3.6^{a}	2.7^{a}	3.1^{a}				
	Spike	1.6^{a}	1.7^{b}	7.7^{a}	9.1^{a}	3.4^{a}	3.4^{a}	4.5^{a}	5.3^{a}				
	Grain	2.6^{ab}	2.3^{a}	18.7^{a}	24.6^{b}	4.3^{a}	4.4^{a}			1.3^{a}	1.9^{b}	0.035^{a}	0.041^{a}
**2012**	Flag leaf	-	3.0	-	4.3^{b}	-	3.4	-	3.0				
	Peduncle	1.4^{a}	1.6^{b}	2.0^{a}	2.8^{b}	2.6^{a}	3.5^{a}	-0.2^{a}	1.9^{b}				
	Glumes	1.4^{a}	1.4^{a}	1.2^{a}	1.2^{a}	2.6^{a}	3.1^{a}	1.0^{a}	2.9^{b}				
	Awns	1.3^{a}	1.7^{b}	1.9^{a}	2.6^{b}	2.9^{a}	3.1^{a}	0.6^{a}	3.1^{b}				
	Grains	2.4^{a}	2.2^{a}	14.7^{a}	16.1^{a}	3.9^{a}	4.4^{b}			2.5^{a}	2.7^{a}	0.036^{a}	0.070^{b}

	Potential organ N contribution											
	2010				2011							
	Flag		Spike		Flag		Peduncle		Glumes		Awns	
Regallo	0.16	a	0.58	ab	0.16	a	0.15	a	0.07	ab	0.15	abc
Anton	0.17	a	0.56	ab	0.18	a	0.21	ab	0.09	ab	0.15	abc
Sula	0.15	a	0.53	ab	0.21	a	0.18	ab	0.07	ab	0.14	ab
Bolo	0.20	a	0.46	ab	0.23	a	0.14	a	0.07	ab	0.16	abc
Don Pedro	0.14	a	0.40	a	0.25	a	0.14	a	0.06	a	0.12	ab
Blanqueta	0.22	a	0.72	b			0.27	b	0.08	ab	0.16	abc
Negro	0.23	a	0.57	ab			0.28	b	0.11	b	0.24	c
Griego de Baleares	0.16	a	0.53	ab			0.28	b	0.07	ab	0.14	ab
Jerez	0.23	a	0.71	b			0.25	ab	0.10	ab	0.10	a
Forment de Artes							0.15	a	0.11	b	0.21	bc
**Level of significance**												
Genotype	0.561		0.17		0.348		0.151		0.22		0.411	

Table S4. Average per genotype of the potential N contribution of the flag leaf blade, spike, awns, glumes and peduncle to the nitrogen accumulated in the grains. Values were calculated as the product of nitrogen content (N content) of the different organs multiplied by their respective dry weight and standardized by the total nitrogen content of mature kernels per spike (Grain N·spike^{-1}). For the total spike N calculation, total grain N of developing grains was subtracted from the calculation (see Materials and Methods). Ten durum wheat genotypes and three replicates per genotype (5 modern cultivars for SI and RF conditions and 5 old cultivars under RF conditions alone, totalling 84 plots in 2011 and 90 in 2012 as the genotype Forment de Artes was discarded due to late phenology in 2011 and old cultivars were discarded in SI in 2010 and 2011) were considered combining rainfed N fertilized (RF+HN) and non-fertilized conditions (RF-LN) and supplemental irrigation N fertilized (SI+HN) and non-fertilized conditions (SI-LN). The experiment was performed under field conditions at the INIA's Experimental Station, Aranjuez, Spain during the 2011 and 2012 growing seasons. Mean values across organs and different growing conditions are significantly different according to the Tukey's honestly significant difference test (P<0.05).

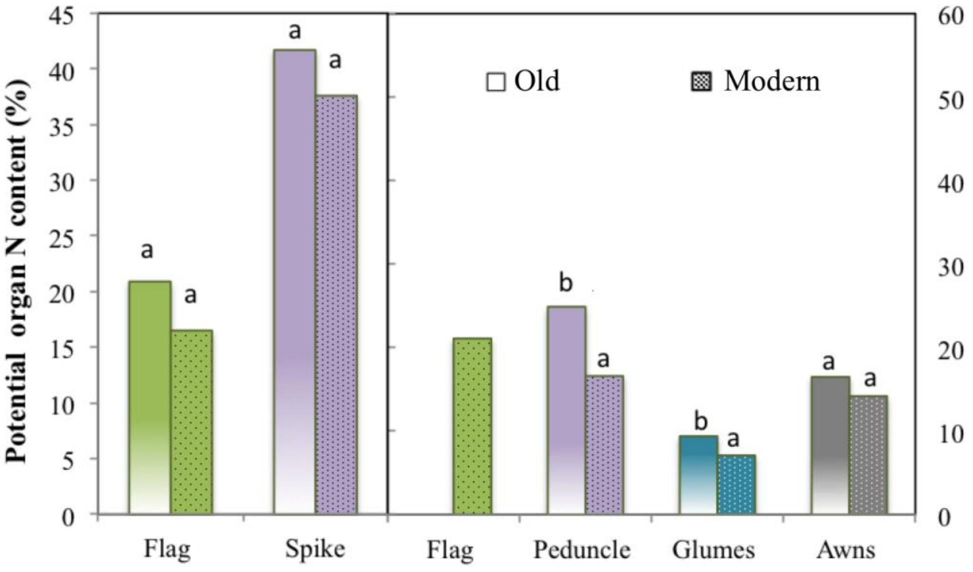

Fig. S1 Potential N contribution of the flag leaf blade, peduncle, spike, glumes and awns to the nitrogen accumulated in the grains separated by modern and old cultivars. Values were calculated as the product of nitrogen content (N content) of the different organs multiplied by their respective dry weight and standardized by the total nitrogen content of mature kernels per spike (Grain N·spike^{-1}). For the total spike N calculation, total grain N of developing grains were subtracted from the calculation (see Material and Methods). Five modern cultivars (60 plots in 2011 and 2012) and five old cultivars (24 plots in 2011, 30 plots in 2012) of durum wheat genotypes (old cultivars were discarded in SI in 2011 and 2012) and three replicates per genotype were considered combining all growing conditions. The experiment was performed under field conditions at the INIA's Experimental Station, Aranjuez, Spain during 2011 (left panel) and 2012 (right panel) growing seasons. Mean values across organs and different growing conditions are significantly different according to the Tukey's honestly significant difference test (P<0.05).

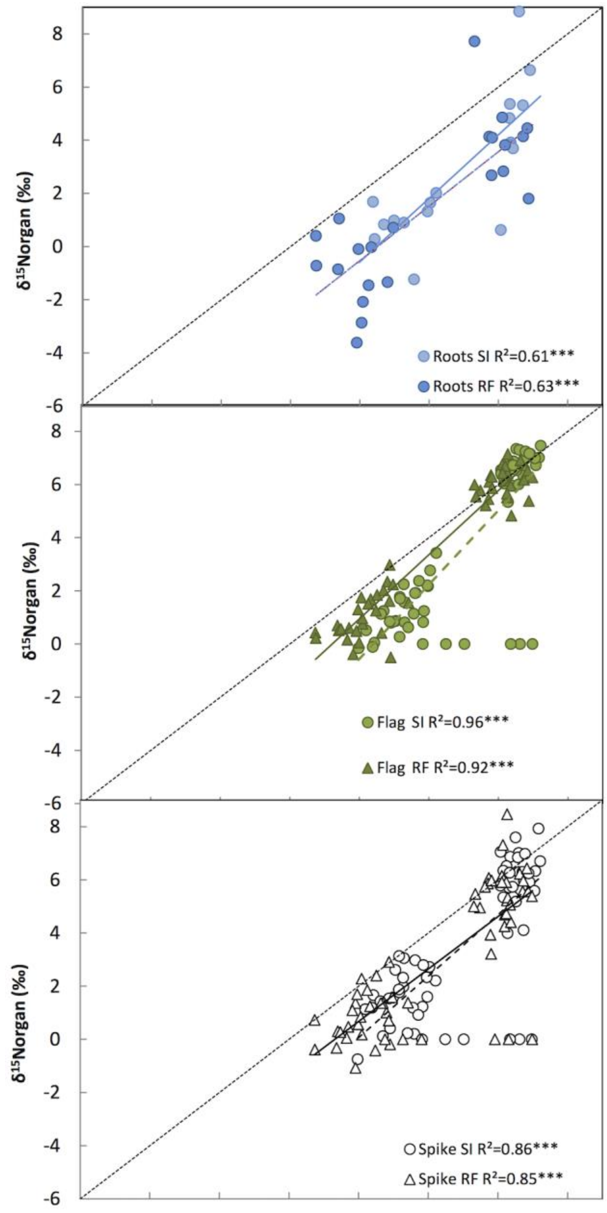

Fig. S2 Linear regression of the relationship between the nitrogen isotope composition ($\delta^{15}N$) in the mature grains (δ^{15}Ngrain) against the $\delta^{15}N$ in the dry matter (DM) of the roots, flag leaf blade and spike. Nine durum wheat genotypes and three replicates per genotype (108 plots) were considered including the four growing conditions (RF+HN, RF-LN, SI+HN and SI-LN) tested at the INIA's Experimental Station, Aranjuez, Spain in 2011. Level of significance: ***, $P < 0.001$; **, $P < 0.01$; ns, $P > 0.05$.

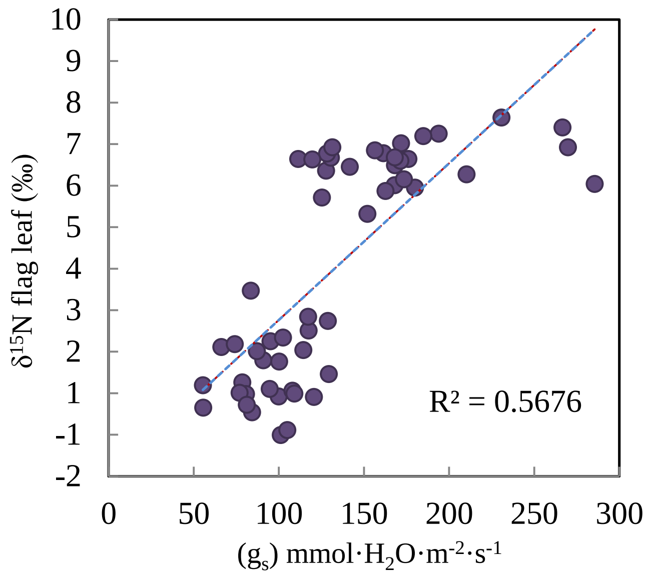

Fig. S3 Linear regression of the relationship between the nitrogen isotope composition ($\delta^{15}N$) in the flag leaf ($\delta^{15}N$) against the stomatal conductance (g_s) under rain fed conditions. Nine durum wheat genotypes and three replicates per genotype (54 plots) were considered including the two growing conditions (RF+HN, RF-LN) tested at the INIA's Experimental Station, Aranjuez, Spain in 2011. Level of significance: ***, $P < 0.001$.
